# 

**Published:** 1884-03

**Authors:** 


We have only space to mention the following books and pamphlets received. At a future time we hope to make a more extended notice of some of them:

*Transactions of the Dental Society of the State of New York.* Fourteenth and fifteenth annual meetings, 1882–1883.

*Transactions of the Ohio State Dental Society.* Eighteenth annual meeting, 1883.

*Woman.* An Oration delivered before the American Academy of Dental Science, Boston. By Norman W. Kingsley, D. D. S.

*Micrometry.* Report of Prof. W. A. Rogers on the Standard Micrometer, Scale A, 1882.

*A Consideration of the Merits and Claims of Artificial Crown- and Bridge-Work.* By J. L. Williams, New Haven, Conn.

*Annual Address delivered before the American Academy of Medicine at New York.* By Henry O. Marcy, M. D., President of the Academy.

*The Pathology and Radical Cure of Hay Fever, or Hay Asthma.* By John O. Roe, M. D., Rochester, N. Y.

*Internal Œsophagotomy.* By John O. Roe, M. D., Rochester, N. Y.

*Discussion of the Utility or Non-Utility of Local Applications in Chronic Catarrhal Laryngitis.* By John O. Roe, M. D., Rochester, N. Y.

*Infusion of Jequirity in Inverterate Pannus.* By Edward S. Peck, M. D., New York.

*Concerning Records.* By Geo. L. Parmele, M. D., D. M. D., Hartford, Conn.

*Micro-Organisms the Essential Factor in Dental Caries.* By C. T. Stockwell, Springfield, Mass.

*On the Relations between Dental Lesions and Diseases of the Eye.* By Henry Power, M. B., London, England.

*Surgical Operations on the Pelvic Organs of Pregnant Women.* By Matthew D. Mann, M. D., Buffalo, N. Y.

*Treatment of Fracture of the Jaw.* By Thos. Brian Gunning, D. D. S., New York.

*The Reciprocal Attitude of the Medical Profession and the Community.* By Alexander Hutchins, A. M., M. D., Brooklyn, New York.

*Zahnarztliche Belehrungen fuer Laien.*

(Dental Instructions for the Laity.)

By Dr. Adolf Petermann, Frankfort-on-the-Main.

*Keine Narkosen—ohne Zeugen.*

(No Anæsthesia without Witnesses.)

By Dr. Adolf Petermann, Frankfort-on-the-Main.

*Zahnarztlicher Verein zu Frankfurt am Main. Officieller Bericht ueber die Festsitzungen.*

(Official Report of the Dental Association of Frankfort-on-the-Main. May Meeting, 1881.)

*The same for May*, 1882.

### The Independent Practitioner—Vol. V.

### PUBLISHED BY DENTISTS FOR DENTISTS.

### Prospectus.

THIS JOURNAL is published by an association of professional men who have no ulterior objects other than to afford to the profession an independent, outspoken Journal, which shall be the exponent of higher aims and a better practice.

Having no connection with any school, clique, or advertising firm, it will be impartial in its judgment of professional matters, and its appeals for support are directed to those who believe that a liberal profession should have an independent literature.

While not neglecting practical details, it will pay especial attention to those oral diseases that have usually been under the care of the general practitioner, but which more properly belong to the specialty of dentistry.

Its pages will be open to any member of the profession who has anything of interest to communicate, and it invites a free expression of views on all subjects medical or dental.

As each of the gentlemen forming the association is practically a member of the editorial corps, it will have abundant opportunity for collecting all the latest professional news and intelligence.

It will have a body of regular contributors, selected from the very best writers in the profession, and will thus be certain of an abundance of interesting original matter.

It will pay especial attention to reports of the meetings of dental societies, and its aim will be to publish an epitome of all important discussions of professional matters.

It will contain original abstracts and translations of all that is of moment in the foreign journals.

Its profits, if any, will be devoted to its improvement by adding needed illustrations, paying for valuable original communications, and enlarging its size whenever this becomes advisable.

To the accomplishment of the work thus outlined (necessarily relying upon the hearty approval of an appreciative profession), each of the publishers of the INDEPENDENT PRACTITIONER has pledged his best efforts.

Editorial communications should be addressed to DR. W. C. BARRETT, No. 11 West Chippewa St., Buffalo, N. Y. Send all business letters to DR. WILLIAM CARR, 35 West Forty-Sixth Street, New York.

| | | |
|---|---|---|
| ABBOTT, FRANK....New York. | CARR, WILLIAM..New York. | JARVIE, WM. Jr..Brooklyn. |
| BARRETT, W. C.........Buffalo. | FRANCIS, C. E....New York. | NORTHROP, A. L.New York. |
| BODECKER, C. F. W. New York. | HILL, O. E...........Brooklyn. | PALMER, S. B......Syracuse. |

